# Neurofeedback-induced facilitation of the supplementary motor area affects postural stability

**DOI:** 10.1117/1.NPh.4.4.045003

**Published:** 2017-11-10

**Authors:** Hiroaki Fujimoto, Masahito Mihara, Noriaki Hattori, Megumi Hatakenaka, Hajime Yagura, Teiji Kawano, Ichiro Miyai, Hideki Mochizuki

**Affiliations:** a Morinomiya Hospital, Neurorehabilitation Research Institute, Osaka, Osaka, Japan; b Osaka University Graduate School of Medicine, Department of Neurology, Suita, Osaka, Japan; c Kawasaki Medical School, Department of Neurology, Kurashiki, Okayama, Japan

**Keywords:** near-infrared spectroscopy, supplementary motor area, rehabilitation, neurofeedback, postural control

## Abstract

Near-infrared spectroscopy-mediated neurofeedback (NIRS-NFB) is a promising therapeutic intervention for patients with neurological diseases. Studies have shown that NIRS-NFB can facilitate task-related cortical activation and induce task-specific behavioral changes. These findings indicate that the effect of neuromodulation depends on local cortical function. However, when the target cortical region has multiple functions, our understanding of the effects is less clear. This is true in the supplementary motor area (SMA), which is involved both in postural control and upper-limb movement. To address this issue, we investigated the facilitatory effect of NIRS SMA neurofeedback on cortical activity and behavior, without any specific task. Twenty healthy individuals participated in real and sham neurofeedback. Balance and hand dexterity were assessed before and after each NIRS-NFB session. We found a significant interaction between assessment periods (pre/post) and condition (real/sham) with respect to balance as assessed by the center of the pressure path length but not for hand dexterity as assessed by the 9-hole peg test. SMA activity only increased during real neurofeedback. Our findings indicate that NIRS-NFB itself has the potential to modulate focal cortical activation, and we suggest that it be considered a therapy to facilitate the SMA for patients with postural impairment.

## 1 Introduction

For patients with neurological diseases, impaired neural networks can cause functional deterioration. Despite recent advances in neurological treatments, rehabilitation remains the most effective and practical treatment option for these patients. Indeed, studies suggest that rehabilitation induces plastic changes in structural and functional neural networks and yields functional recovery.^1^ Accordingly, facilitating plastic reorganization of neuronal networks via neuromodulation is a therapeutic strategy for augmenting functional recovery. Several methods of neuromodulation have been introduced to the field of rehabilitation; these include repetitive transcranial magnetic stimulation, transcranial direct-current stimulation, and neurofeedback.^2^ While each of these techniques noninvasively affects local cortical excitability, neurofeedback using neuroimaging tools has drawn particular attention because it does not require external stimulation.^3^^,^^4^ As with other neuromodulatory techniques, studies have shown that the effects of neurofeedback depend on the cortical function of the local target cortical region. For instance, neurofeedback studies using fMRI have shown that suppressing activity in the anterior cingulate cortex enabled sensory control of pain^5^ and that activating the premotor cortex (PMC) increased the rate of finger tapping.^6^ Several studies have adopted fMRI as a tool for applying region-specific neurofeedback because of its high special resolution. However, while it can be used for experiments, it is not feasible for clinical application because it requires huge equipment and puts onerous constraints on patients. Therefore, we developed a clinically feasible near-infrared spectroscopy-mediated neurofeedback (NIRS-NFB) system^7^ and reported that lateral PMC facilitation by NIRS-NFB combined with mental practice improved upper-limb function after stroke.^8^ However, several issues require clarification for clinical application of NIRS-NFB systems. Because our previous study applied NIRS-NFB concurrently with a motor imagery task, we were unable to determine whether neuromodulative facilitation using NIRS-NFB results directly in functional recovery or if the process improves the quality of motor imagery,^7^ which in turn enhances functional recovery and cortical activation. Additionally, it remains unclear whether region-specific facilitation by NIRS-NFB has distinct behavioral effects related to the function of the facilitated cortical region.

Studies suggest that a widely distributed neuronal network that includes the lateral PMC and supplementary motor area (SMA) is involved in motor-related plastic reorganization, which is associated with functional recovery.^9^^,^^10^ The SMA is thus another potential target for therapy using neurofeedback. However, given that the effect of neurofeedback depends on local cortical function, and because the SMA contributes to many aspects of motor control,^11^ the effect of facilitating the SMA via neurofeedback is ambiguous. Human and animal studies indicate that the SMA contributes to normal gait and postural control,^12^^–^^14^ gross trunk and limb movement,^15^^,^^16^ motor planning,^17^ interlimb coordination,^18^ sequential ordering of complex movements,^19^ and self-initiating movement.^20^ These varying functions suggest that SMA facilitation might improve postural control, truncal movement, or even precise upper-limb movement. To help determine the most appropriate use of NIRS-NFB intervention for augmenting motor recovery, we investigated the behavioral effect of facilitating the SMA via NIRS-NFB. We applied SMA-targeted NIRS-NFB to healthy participants without imposing any additional task and assessed multiple behavioral measurements, including center of pressure (COP) for postural instability and ability on the 9-hole peg test for hand dexterity.

## 2 Materials and Methods

### 2.1 Participants

We obtained written informed consent from 20 healthy adult participants who had no history of neurological disorders (7 men; mean age: 28.1±4.6  years). Handedness was determined by the Edinburgh Handedness Inventory,^21^ and all participants were right-handed. The study was approved by the Ethics Committee of Morinomiya Hospital and conducted in accordance with the Declaration of Helsinki.

### 2.2 Near-Infrared Spectroscopy Measurements and Detector Channel Setting

**Fig. 1:**
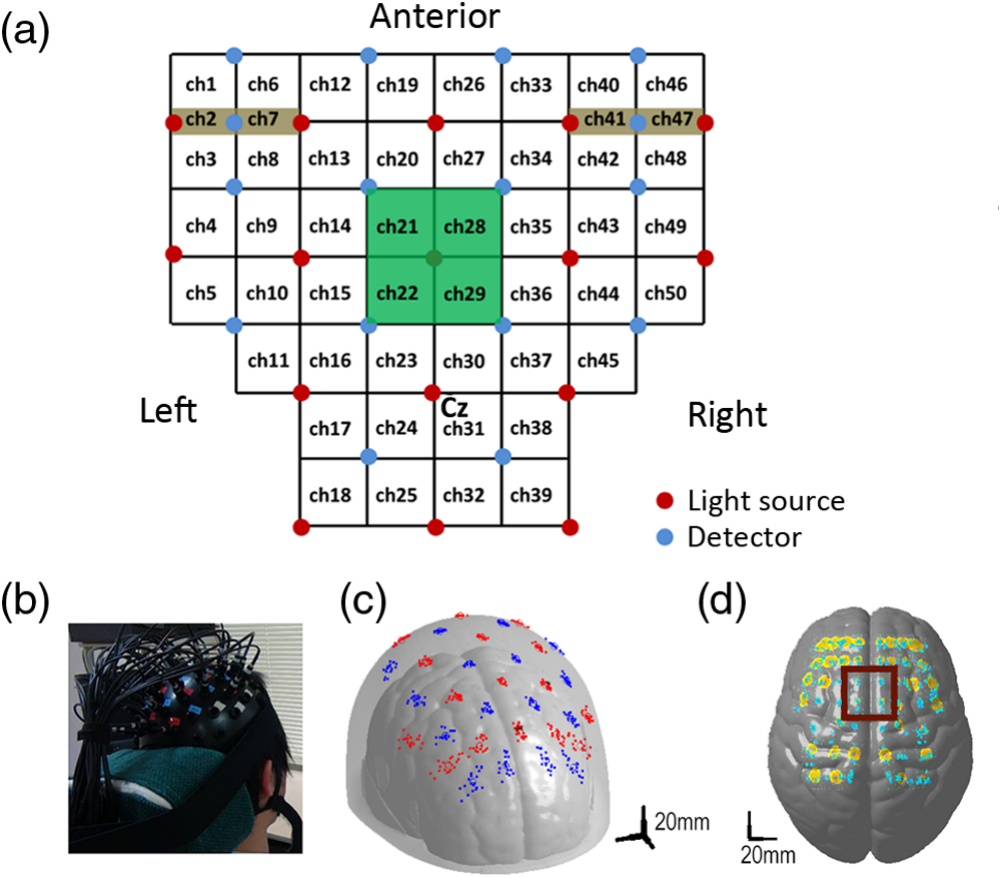
Cortical registration of the NIRS channels. (a) Arrangement of the optodes on the frontoparietal scalp with an interoptode distance of 3 cm, with four short-distance channels. The light source at the center of the third row was placed at Cz. Four channels (21, 22, 28, and 29) cover the SMA. (b) We used a custom-made hard-plastic holder to hold the fibers tightly to the scalp. (c) The location of each optode was transferred to the standard MNI space using the affine transformation matrix, calculated with SPM5 software. Red dots indicate the light sources and blue dots indicate the detectors for each participant. (d) Estimated location of each NIRS channel as the midpoint of the corresponding light source–detector pair and adoption of the balloon-inflation method. The yellow area represents the dispersal area for the possible cortical projection points (the average + 1 SD of the estimated points). Cyan dots represent the cortical projection points of the NIRS-mediated channels for each participant.

We used a continuous wave NIRS system with 16 light sources and detectors (OMM-3000; Shimadzu Corp., Kyoto, Japan) to detect changes in cortical hemodynamics. Optodes were placed on the frontoparietal scalp using a custom-made hard-plastic holder with an interoptode distance of 3 cm [Figs. 1(a) and 1(b)]. The light source at the center of the third row was placed at the vertex (Cz) for each participant [Fig. 1(a)]. The NIRS channel was defined as the midpoint of the corresponding light source–detector pair [Fig. 1(a)]. Cortical activity was measured from 50 channels at 4 Hz, with four short-distance channels on the bilateral prefrontal scalp [Fig. 1(a)] to eliminate contamination of the NIRS signal by scalp blood flow.^22^ We applied a modified Beer–Lambert law^23^ to calculate signal changes derived from oxygenated hemoglobin (OxyHb) and deoxygenated hemoglobin (DeoxyHb) using absorption changes in infrared light at 780, 805, and 830 nm. Changes in the hemoglobin-derived signal were measured in an arbitrary unit (mmol/L×mm). Similar to our previous studies,^7^^,^^8^ we estimated the position of each channel using individual anatomical three-dimensional T1-weighted magnetic resonance (MR) images from all participants except for one participant for whom MR images could be obtained. The spatial configuration of the optodes on the scalp was maintained using a virtual holder set. Raw head images were normalized to the standard ICBM152 (ICBM)^24^ template using SPM8 software,^25^ and the location of each optode was estimated on the Montreal Neurological Institute (MNI) standardized scalp via an affine transformation for normalization. Across participants, we used the balloon-inflation method^26^ to calculate the mean coordinates for each optode and the cortical projection point from each NIRS channel. The averaged coordinates and their variance were estimated using individual normalized data. Because the dispersion of the estimated channel positions for each participant was within several millimeters [Figs. 1(c) and 1(d)] and considering the relatively poor spatial resolution of NIRS (several centimeters), we assumed that each channel location was similar across participants. We estimated the center position of the cortical region covered by each channel using MRIcro software,^27^ which provided Brodmann areas and automated anatomical labeling.^28^

### 2.3 Signal Processing for Real-Time Neurofeedback

**Fig. 2:**
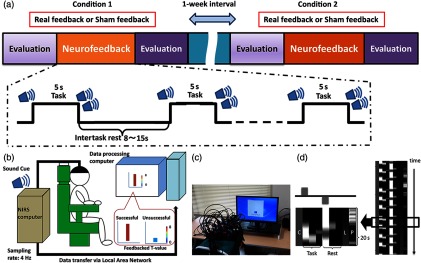
Task protocol and NIRS-mediated neurofeedback system. (a) Neurofeedback was applied in the real- and sham-FB conditions on different days with a >1-week interval. Each session comprised 16 repetitions of a 5-s trial with pseudorandomized rest periods between 8 and 15 s. The order of the task conditions was counterbalanced across participants, who were blind to the task conditions. (b) Participants were asked to raise the FB bar after an auditory cue without any suggestions about how to accomplish this. Successful trials exhibited higher sustained FB values, even in the rest periods, in accordance with the FB t-value bar. (c) The NIRS-NFB system in use and user-interface for neurofeedback task. (d) The design matrix for the real-time sliding-window GLM analysis. The time window was 80 data points wide (20 s). The matrix consisted of one constant column (C) three columns (a hemodynamic response function and its temporal and dispersion derivatives) for the task and rest phases, respectively, one linear term (L), and a primary component of the short-distance channel data (P). Task-related signal changes were estimated as a β-value comparing the task data against the resting data.

Our neurofeedback system consisted of an NIRS system, a computer for data analysis, and a feedback (FB) monitor, as previously reported^7^^,^^8^ [Figs. 2(b) and 2(c)]. Based on previous findings, we used OxyHb-derived signals as the index of brain activity,^7^ and real-time analysis of the NIRS signal was performed according to previously described methods.^7^ Briefly, hemoglobin signals were measured at a sampling rate of 4 Hz, and these data were processed by the NIRS computer and transferred to a data-processing computer via a local area network cable [Fig. 2(b)]. To avoid the effect of obvious motion artifacts or the unstable attachment of the optodes to the scalp, we visually inspected all the raw NIRS signals throughout the experiments. Task-related changes in the signal were estimated from the most recent 20 s of data using an adaptive general linear model (GLM) analysis with least-squares estimation. The observation window was measured for 20 s at 4 Hz, contained 80 data points, and covered at least one trial block [Fig. 2(d)]. To eliminate extracortical contamination, such as the influence of respiration, heart rate, and motion artifacts on scalp blood flow, we simultaneously performed a principal component analysis using data from the short-distance channels and included the primary principal component as a regressor in the model. Analyses were performed using in-house software running on MATLAB^®^ (R2012b; MathWorks, Natick, Massachusetts). We calculated β-coefficients and t-values for the channel covering the target cortical area as indices of task-related local cortical activation. Based on the standardized MNI coordinates of the channels, we selected four channels (21, 22, 28, and 29) as those covering the SMA [Fig. 1(a)]. We used the maximum t-value from these four channels as the FB value, which was reflected in the height and color of the vertical FB bars that were shown to the participants [Figs. 2(b) and 2(c)]. Because our online analysis algorithm evaluated the contrast between signals in the task and rest periods, the FB bar did not represent the absolute signal. Rather, it was the result of an online statistical analysis based on the signals obtained from the target channels. As in our previous NIRS-NFB studies,^7^^,^^8^ we told participants that sustained higher FB values represented successful trials and that they should try to keep the height and color of the bar at elevated levels, even in the rest task (see Sec. 2.4 for details).

### 2.4 Task

Participants sat comfortably with their eyes open in an armchair that had a headrest. The FB task comprised 16 repetitions of a 5-s trial, with pseudorandomized rest periods ranging from 8 to 15 s between each task period [Fig. 2(a)]. The start and end of each trial were signaled with audio cues (start, single beep and end, double beep). Participants were asked to increase their cortical activity during the task periods and to relax during the rest periods, which would eventually lead to decreased cortical activity in the target area. As in our previous NIRS-NFB studies,^7^^,^^8^ task-related signal changes were estimated by comparing β-coefficients between task and resting data using one-sided (right-tailed) paired t-tests. The calculated t-values were used as markers of cortical activation at each channel. The largest of the calculated t-values for the four channels covering the SMA (21, 22, 28, and 29) was shown as the height and color of the vertical FB bar to provide FB for the participants. As stated above, because our system evaluates the cortical activation contrast between rest and task, the FB value becomes higher when the difference between the two is greater (i.e., cortical activation increases during the task and decreases during rest). Therefore, relaxing during the rest period raised the level of the FB bar. Generally, the color change in the FB bar was recognizable when a t-value of 2 was attained [Figs. 2(b) and 2(c)], although the threshold values were not presented to the participants. To eliminate possible interference from motor activity in the lower-limb muscles and their afferent input, we monitored muscle activity from the anterior tibial and soleus muscles by surface electromyogram (EMG) at 1000 Hz. Participants were not given any instructions or suggestions for specific strategies that would help them complete the task, such as using motor execution or motor imagery. Each individual participated in two sessions that were separated by at least 1 week. In one session, their cortical activation was evaluated by a real-time analysis algorithm and provided as the FB value (the real condition), while in the other session, FB values were calculated using prerecorded cortical activity data from other individuals that did not participate in the task (the sham condition). The prerecorded data were obtained from the current study as well as a preliminary study using a similar task. In the sham condition, the prerecorded data were randomly selected from these pooled data, which include both the real and sham conditions. In the sham condition, the FB value did not represent the participants’ own cortical activation and did not reflect any effort that might cause a cortical activation change. Therefore, we predicted that no substantial neuromodulation effect would occur in the sham condition. The order of the two conditions was counterbalanced across participants, and all participants were blinded to the conditions [Fig. 2(a)]. Immediately after each neurofeedback session, we administered a self-assessment questionnaire that asked the participants how well they were able to concentrate on the task (Likert-scale: higher value = better concentration) (Table 1), but we did not provide any suggestions about cognitive strategy.

**Table 1:** Self-assessed concentration scores in 20 participants for both real and sham conditions.

Self-assessment score			
Subject	Real FB	Sham FB	Interval (days)
1	4	4	16
2	5	5	86
3	5	5	21
4	5	4	9
5	5	5	19
6	5	5	17
7	4	5	142
8	5	5	9
9	5	4	7
10	4	4	90
11	4	5	21
12	5	5	35
13	4	5	9
14	5	5	65
15	4	5	50
16	5	5	32
17	5	5	22
18	5	5	42
19	4	4	7
20	4	4	8
Average	4.6	4.7 ns	35.4

Note: Self-assessment score: 5: excellent, 4: good, 3: average, 2: fair, 1: poor.

ns: Not significant.

### 2.5 Behavioral Measures

To evaluate the behavioral effect of neurofeedback, we took several measurements before and after both NFB sessions. To assess postural control, we asked participants to maintain a stable upright position for 30 s with their feet together and eyes open and then again for 30 s with their eyes closed. We recorded the total trajectory of the COP at 50 Hz, using a tactile sensor sheet (BIG-MAT™; Nitta Corp., Osaka, Japan). The value was calculated using the formula shown below, with lower values indicating less postural sway during standing (i.e., thus better postural control). Mean COP values from the 30-s eyes-open and eyes-closed conditions were used as measures of postural control

$${\mathrm{COP}}_{\text{length}}=\overset{n-1}{\underset{i=1}{\sum }}\sqrt{{\left(X_{i+1}-X_{i}\right)}^{2}+{\left(Y_{i+1}-Y_{i}\right)}^{2}}.$$

To assess upper-limb function for sequential and fine motor control, participants performed the 9-hole peg test using their nondominant hands. This test is a simple and reliable method for evaluating finger dexterity in healthy individuals,^29^ and SMA involvement has been suggested to be involved in rehabilitation-related improvement on this task after stroke.^30^ Following several practice sessions, participants performed the task three times, and mean scores were calculated for further analysis.

We used repeated-measures analyses of variance (ANOVAs) to assess postural control and upper-limb function, with the FB condition (real/sham) and assessment periods of the behavioral task (pre/postneurofeedback session) as the within-subject factors. Statistical significance was set at p<0.05 with Bonferroni correction.

We also performed a paired t-test comparing the average FB values that were provided to participants as the height and color of the vertical FB bar. Statistical significance was set at p<0.05.

### 2.6 Off-line Image Analysis

NIRS data were analyzed using a GLM via an in-house program running on MATLAB^®^.^13^ The preprocessing procedure included removing baseline drift with a high-pass filter (cutoff frequency=0.01  Hz). To estimate the effect of neurofeedback on cortical activity, we divided the 16 trials into two blocks and made channel-based intraparticipant contrast images that compared task-related cortical activity among blocks. The first 6 trials comprised an early block, and the last 10 trials were the late block. The division among analysis blocks was made after the sixth trial because healthy individuals reach a plateau at the fifth or sixth trial of motor-related tasks.^31^ Thus, the contrasts for detecting the effect of immediate neuromodulation on cortical activation were as follows: (${\text{Real}}_{\text{Late}}-{\text{Real}}_{\text{Early}}$) and (${\text{Sham}}_{\text{Late}}-{\text{Sham}}_{\text{Early}}$). Next, we performed a second-level group analysis that adopted a random-effects model. Individual contrasts were used as the dataset, and two-tailed one-sample t-tests were performed against a mean of zero. Statistical significance was set at p<0.05 (false discovery rate-corrected for multichannel recording of cortical activation). To confirm the effect of neurofeedback on SMA activation, we also performed a timeline analysis of the left SMA (Ch. 21 and 22) with repeated-measures ANOVA to assess the interaction between task block (early or late) and the conditions. Statistical significance was set at p<0.05 with Bonferroni correction.

## 3 Results

NIRS-NFB did not lead to any adverse effects, and all participants completed both the real and sham conditions. The mean interval among sessions was 35.4±36.2  days. No participants reported difficulty in understanding or concentrating on the task. EMG analysis indicated that participants did not move at all during task periods.

**Fig. 3:**
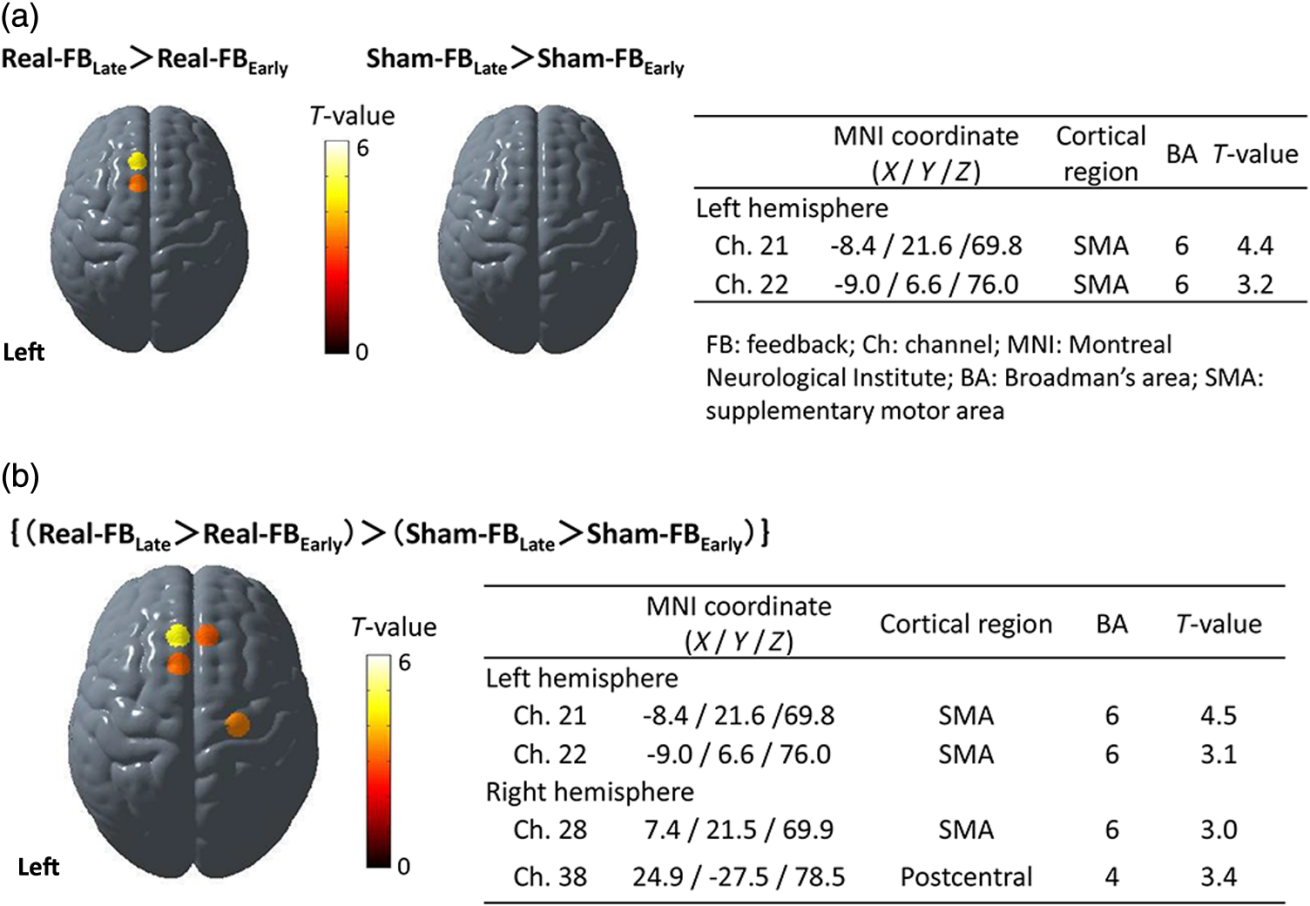
Changes in cortical activity associated with neurofeedback. (a) Multiparticipant analysis showed that the activity in the left SMA was significantly higher after real neurofeedback but not after sham neurofeedback. (b) Temporal changes in cortical activity were more prominent during real neurofeedback in both the right and left SMA compared with sham neurofeedback.

**Fig. 4:**
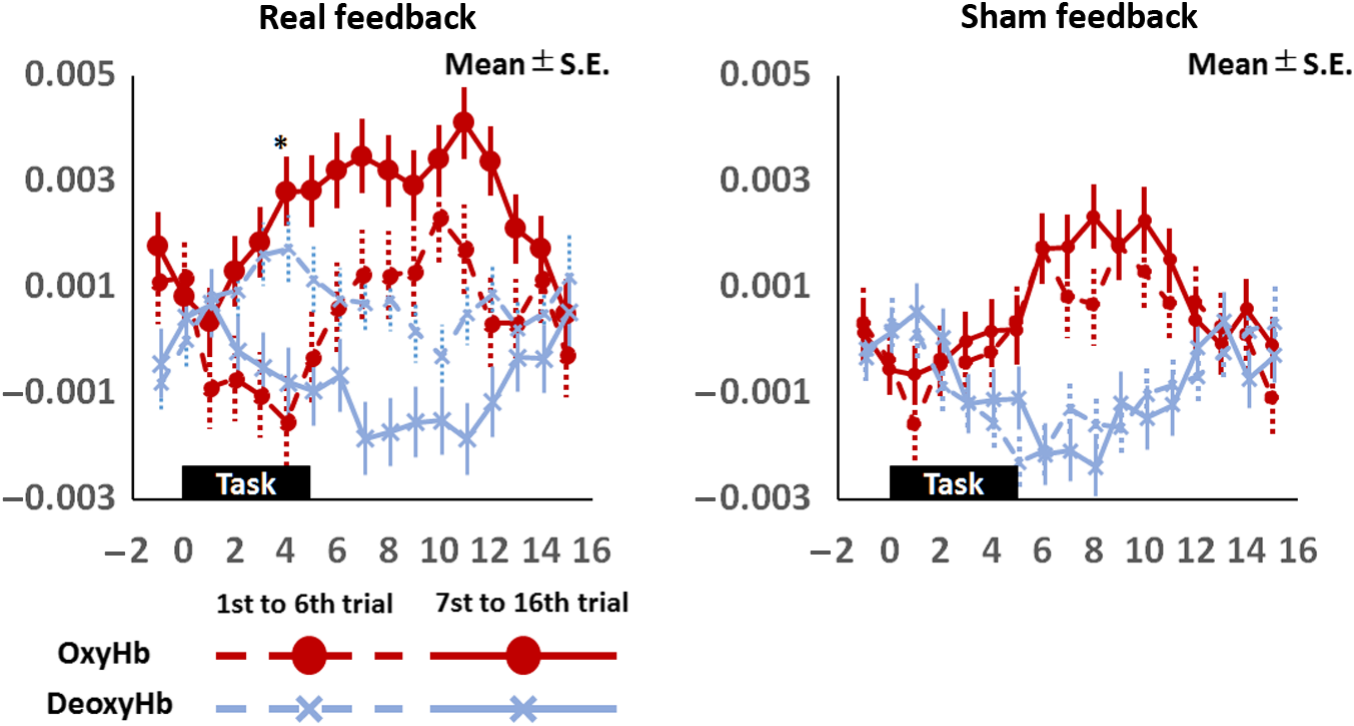
Comparison between early and late trials. Comparison between early and late trials (trials 1 to 6 versus trials 7 to 16) revealed a significant increase in the task-related OxyHb signal only during real neurofeedback and a significant interaction between time and condition. Posthoc analysis revealed that the significant increase in the OxyHb signal during real neurofeedback occurred 4 s after task onset (*).

Multiparticipant analysis of the real and sham conditions revealed significant facilitation of the left SMA only in the real condition [Fig. 3(a)]. Similarly, the comparison between early and late trials (trials 1 to 6 versus trials 7 to 16) (Fig. 4) revealed a significant immediate neuromodulation effect on the OxyHb signal only in the real condition ($F_{1,2558}=5.86$, p<0.05) with a significant interaction between time and task conditions ($F_{16,2543}=2.14$, p<0.005). Bonferroni posthoc analysis revealed a significant increase in the OxyHb signal 4 s after task onset ($t_{2558}=4.0$, p<0.001). The OxyHb signal did not differ significantly between early and late trials during the sham condition nor did the DeoxyHb signals in either condition. Additionally, comparing the amount of pre/postchange between conditions showed more prominent facilitation in the bilateral SMAs during the real condition than during the sham condition [Fig. 3(b)].

**Fig. 5:**
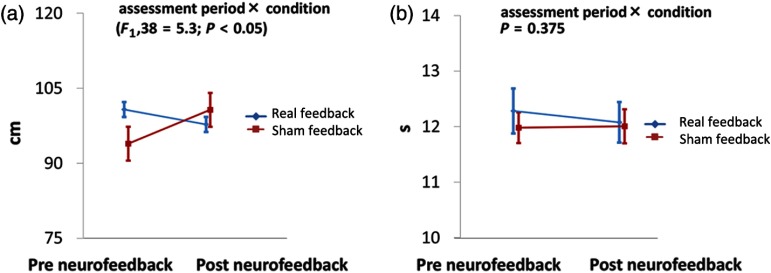
Assessment of postural stability and upper-limb function. (a) Effect on the COP length. A significant interaction was found between condition (real/sham) and assessment period (pre/postneurofeedback). The vertical line indicates the COP trajectory. (b) Effect on 9-hole peg test performance. No changes were observed after neurofeedback intervention in either condition, and no significant interaction between condition and assessment period was found. This indicates that the neurofeedback did not affect upper-limb function. The vertical line indicates the timing of the 9-hole peg test.

Assessment of postural control using total trajectory of the COP revealed no main effect of condition (real versus sham: $F_{1,38}=0.034$; p=0.855) or assessment period (pre versus post: $F_{1,38}=0.795$; p=0.378). Importantly, we found a significant interaction between condition and assessment period [$F_{1,38}=5.3$; p=0.027; Table 2, Fig. 5(a)]. Bonferroni posthoc analysis did not reveal any significant differences in COP, either between conditions (prereal versus presham: $F_{1,19}=4.1$; p=0.056; postreal versus postsham: $F_{1,19}=0.4$; p=0.518) or between assessment periods (pre versus post: $F_{1,19}=1.7$; p=0.206; presham versus postsham: $F_{1,19}=3.6$; p=0.073). Similarly, we found no significant main effects of condition or assessment period on hand dexterity (9-hole peg test; condition: $F_{1,19}=1.702$; p=0.208; assessment period: $F_{1,19}=0.099$; p=0.756). However, unlike the COP, hand dexterity showed no significant interaction between condition and assessment period [$F_{1,38}=0.8$; p=0.375; Table 2, Fig. 5(b)].

**Table 2:** Measures of postural stability and upper-limb function in 20 participants for both real and sham neurofeedback.

		Real FB	Sham FB
${\mathrm{COP}}_{\text{length}}$ (cm)*	Pre	100.8±36.7	93.9±29.9
	Post	97.8±34.9	100.7±35.6
9-hole peg test (s)	Pre	12.3±1.6	12.0±1.1
	Post	12.1±1.5	12.0±1.2

Note: COP: Center of pressure. Data are shown in Mean±SD.

* Significant assessment period×condition interaction, p<0.05.

Average FB intensities were significantly higher for the sham condition than for the real condition (p<0.001), suggesting that there was no reward effect in the real condition and no penalty effect in the sham condition.

## 4 Discussion

### 4.1 Neuromodulatory Effect of Near-Infrared Spectroscopy-Mediated Neurofeedback and Concurrent Task During Neurofeedback

Although previous studies using fMRI and other imaging techniques have suggested that neurofeedback can modulate brain activity,^4^ few studies have used NIRS-mediated systems. We previously reported that our NIRS-NFB system could increase motor-related brain activity in healthy and poststroke individuals.^7^^,^^8^ However, because the NIRS-NFB interventions in our previous studies were performed in conjunction with a motor imagery task, we could not rule out the possibility that the major source of cortical facilitation was the efficient motor imagery that accompanied cortical monitoring using NIRS-NFB and that NIRS-NFB by itself had little effect. In addition, reports regarding the effect of a specific strategy concurrently performed during neurofeedback have not been consistent in previous fMRI-mediated neurofeedback studies of SMA activation. One study has proposed that specific strategies—including motor imagery—would increase interindividual variability,^32^ while another has suggested that FB efficacy would be low when specific task suggestions were lacking.^33^ Therefore, we conducted this study to confirm the neuromodulatory effect that NIRS-NFB can induce on its own. The timeline analyses revealed that real neurofeedback facilitated activation in the target cortical area (SMA) even without performing any specific task, suggesting that NIRS-NFB itself had a neuromodulatory effect, similar to the findings reported in a previous neurofeedback study using fMRI.^5^^,^^34^ In addition, we found that the time courses in the early trials were unstable only in the real FB condition. Considering that there was some overlap between pre- and posttask periods in the analysis because the intertask interval was 8 to 15 s in this study, an immediate neuromodulatory effect on cortical activation in the early trials might cause an unstable temporal profile.

### 4.2 Supplementary Motor Area Facilitation and Postural Stability

Although posthoc analysis revealed no significant immediate effects in either condition, our findings revealed a significant interactive effect of neurofeedback condition (real/sham) and assessment period (pre/post) on the postural control measure (COP). In contrast, hand dexterity measures did not show any interaction between the conditions and assessment periods nor any significant main effects.

In hierarchical neural networks for postural control, animal studies indicate that the cerebral cortex is primarily involved in voluntary adjustment or precise control of posture, whereas the brainstem and cerebellum regulate automated postural control.^35^^,^^36^ In contrast, cumulative evidence suggests that human bipedal posture, which is unstable and vulnerable in nature, requires more cortical regulation than postural control in quadruped animals.^11^^,^^37^^–^^40^ Anatomically, the SMA has dense connections with the pontomedullary reticular formation (PMRF), which is believed to regulate truncal muscle tone through the reticulospinal tract.^35^ Neuroimaging studies in humans have also emphasized the importance of the SMA and its projection fibers to the PMRF in gait and postural function,^14^^,^^37^^–^^41^ and other studies have noted SMA involvement in regulating anticipatory postural adjustments^42^^,^^43^ and motor imagery of static and dynamic postural tasks.^44^ These findings suggest a functional correlation between SMA activation and the ability to maintain posture. Our finding that the neurofeedback conditions (real/sham) and assessment periods (pre/post) significantly interacted suggested a functional correlation between SMA activity and postural control.

The significant interaction has several interpretations. First, because the current study included only healthy young participants, significant improvement in postural ability might have been limited by a ceiling effect and, therefore, difficult to identify. Second, our findings showed a near-significant worsening effect on postural control measures in the sham condition, suggesting that sham neurofeedback on SMA might have a deteriorating effect. Considering that NIRS-NFB is a task that demands high levels of concentration, the reduced postural stability might have been caused by the mental fatigue of an unusual task, as previously reported,^45^ and sustained postural stability in the real condition might suggest the potential beneficial effect of the SMA facilitation. Notably, our prior investigation into the clinical efficacy of NIRS-NFB facilitation of the lateral PMC for poststroke upper-limb paresis^8^ did not show any detrimental effects of sham NIRS-NFB. However, we must cautiously consider the possible negative effect of sham NIRS-NFB when conducting double-blind clinical trials with patients who have neurological diseases. Further studies are required to clarify whether, or under what conditions, sham neurofeedback can have negative effects.

### 4.3 Supplementary Motor Area Facilitation and Hand Dexterity

In addition to postural control, the SMA is involved in many other motor control tasks. In particular, reports have emphasized the role of the SMA in complex upper-limb movements. Recent findings have revealed dense anatomical and functional connections between the SMA and the primary motor cortex.^46^^,^^47^ Additionally, enhanced functional connectivity during hand movements^48^ suggests that interactions between the SMA and primary motor cortex might also be important for the fine motor control of unilateral and bilateral upper-limb movements. Behavioral sequences can be represented as combinations of prelearned multidimensional action modules,^49^ and the SMA has been suggested to be involved in memory-guided^19^ self-initiated movement^50^ by selecting appropriate task-relevant action sequences.

Despite this evidence for a correlation between the SMA and upper-limb function, we did not detect any effect of SMA facilitation on hand dexterity. One reason for this could be that, because NIRS cannot detect activation in deeper cortical areas, our system was only able to facilitate the dorsal part of the SMA. This notion is supported by evidence that the dorsal part of the SMA is primarily dedicated to lower-limb function, whereas the rostral and ventral parts are mainly responsible for upper-limb function.^51^ A second possible interpretation is that the task itself did not fully engage the SMA. This might have occurred because the 9-hole peg test uses visuo-spatially guided sequential movements, while studies suggest that the SMA is involved in memory-guided^19^^,^^41^ self-initiated movements,^11^^,^^50^^,^^52^ and the dorsal premotor area controls movements guided by external information.^53^ Therefore, the dorsal premotor area, rather than the SMA, could be a more appropriate target for the 9-hole peg test. A third possibility is that assessment used for hand dexterity was relatively insensitive to changes and was inadequate owing to a ceiling effect. The 9-hole peg test requires precise sequential movements of the fingers to pick up a small peg and insert it into the appropriate hole. Although it requires hand dexterity, this task might be underpowered for detecting effects of neurofeedback in healthy subjects.

### 4.4 Limitations

This study had the following limitations. Regarding the technical aspects of NIRS, several researchers have noted intrinsic contamination by extracortical factors, including motion artifacts and the influence of respiration and heart rate on scalp blood-flow.^22^^,^^54^ Although several methods exist to remove such artifacts, a “gold standard” has yet to be established.^55^^–^^58^ In this study, we addressed this inherent limitation by arranging short-distance channels. Penetration of the near-infrared light has been suggested to depend on the distance between the light emitter and light detector, and short-distance channels primarily represent signals from superficial layers.^59^ Instead of low-pass filtering, we performed a principal component analysis using short-distance channel data and included the primary component into the regression model. This served to eliminate the effects that respiration and heart rate had on task-related changes in scalp blood flow.

Another limitation was that we could not obtain any information about strategy used by the participants. We did not encourage any particular strategy and only instructed participants to raise the FB bar as much as they could through trial and error. Therefore, we cannot completely exclude the possibility that participants covertly used a specific strategy that included motor imagery. Because cognitive load for NFB tasks should affect the NIRS signal,^22^^,^^54^ participants might have overtly or covertly controlled their respiration or muscle contraction during the task to raise the FB bar level. However, we propose that these factors are unlikely to have influenced the neurofeedback effect in this study because we regressed out potential physiological artifacts using the primary component of the short-distance channel data. The lack of a difference in self-assessed concentration between sessions and in the amount of lower-limb movement (EMG activity) also suggests that the attentional load and any muscle contractions were similar between the conditions. As for the specific strategy for NFB in this study, we did not ask participants if they had used a motor imagery strategy because we were concerned that such a question after the first task would lead them to use that strategy in the second session. However, a more precise assessment of strategies would be helpful for understanding interindividual variability and developing the most effective strategy for NIRS-NFB.

Finally, although we assumed that FB signals in the sham FB condition contained no useful information for neuromodulative facilitation, the FB signal itself might have acted as a reward, thereby affecting cortical activation and behavioral function even in the sham condition.^60^ However, we believe this is unlikely because the average FB value in the real condition was significantly lower than that in the sham condition.

## 5 Conclusion

This study confirmed that NIRS-NFB can facilitate focal cortical activity without any concurrent task and suggests that NIRS-NFB can be used as a neuromodulatory tool. The significant interaction between the neurofeedback condition and the assessment period for the balance measures implies a functional correlation between the SMA and postural control. Thus, the SMA could be a possible therapeutic target for augmenting balance recovery via neuromodulatory facilitation. Further studies involving neurological patients are warranted.
